# Proteomic dissection of the *Arabidopsis* Golgi and trans-Golgi network

**DOI:** 10.3389/fpls.2012.00298

**Published:** 2013-01-03

**Authors:** Harriet T. Parsons, Georgia Drakakaki, Joshua L. Heazlewood

**Affiliations:** ^1^Department of Plant and Environmental Sciences, University of CopenhagenCopenhagen, Denmark; ^2^Department of Plant Sciences, University of California at DavisDavis, CA, USA; ^3^Joint BioEnergy Institute, Lawrence Berkeley National LaboratoryBerkeley, CA, USA; ^4^Physical Biosciences Division, Lawrence Berkeley National LaboratoryBerkeley, CA, USA

**Keywords:** Golgi, *trans*-Golgi network, proteomics, LOPIT, free-flow electrophoresis, *Arabidopsis*, SYP61

## Abstract

The plant Golgi apparatus and *trans*-Golgi network are major endomembrane trafficking hubs within the plant cell and are involved in a diverse and vital series of functions to maintain plant growth and development. Recently, a series of disparate technical approaches have been used to isolate and characterize components of these complex organelles by mass spectrometry in the model plant *Arabidopsis thaliana*. Collectively, these studies have increased the number of Golgi and vesicular localized proteins identified by mass spectrometry to nearly 500 proteins. We have sought to provide a brief overview of these technical approaches and bring the datasets together to examine how they can reveal insights into the secretory pathway.

## BACKGROUND

At its simplest level, subcellular proteomics attempts to identify all proteins in a particular compartment. However, even with such a basic definition in mind, the Golgi proteome presents conceptual difficulties; functional proteins in the Golgi may also be functional elsewhere (Ondzighi et al., 2008), whilst endoplasmic reticulum (ER)–Golgi connections (Boevink et al., 1998) makes absolute divisions between the proteomes of these compartments somewhat futile. A number of proteins are known to form functional associations on the cytoplasmic face of cisternae but are part of the cytosol (Ito et al., 2011), so the very definition of the Golgi proteomes is problematic. Furthermore, in such an architecturally heterogeneous organelle, simply identifying all the proteins present in the Golgi is not that helpful unless we can classify them according to sub-Golgi location, post-Golgi compartments, cargo, resident, or dual-localized proteins. The plant Golgi poses a challenge in terms of isolation, not least because of its fragmented morphology. In mammalian cells Golgi stacks tend to be less numerous per cell with fewer, longer cisternae which are less tightly associated with the ER and could be relatively easily isolated (Morre and Mollenhauer, 2009). Excepting highly conserved pathways such as protein *N*-linked glycan processing, few similarities exist between plant and mammalian Golgi. Thus assuming Golgi-residency between the two systems based on homology alone is not possible. Earlier work on Golgi from rat liver was therefore of limited help either in terms of providing an isolation strategy or a comprehensive bank of marker proteins (Taylor et al., 1997). The plant Golgi is much less structurally defined during and after cell homogenization than, for example, plastids or mitochondria. Consequently, quality control of and improvements to isolation strategies have been tricky and therefore purity limited when using sucrose density centrifugation strategies (Morre and Mollenhauer, 1964). In short, it is easy to understand why progress in Golgi proteomics has trailed behind other subcellular compartments in plants. In light of the shortcomings of sucrose density centrifugation for plant Golgi purification, two more technical but very different approaches have been successfully applied, namely localization of organelle proteins by isotope tagging (LOPIT) and free-flow electrophoresis (FFE). The LOPIT approach does not distinguish between Golgi and the *trans*-Golgi network (TGN) localized proteins but identifies resident proteins (Dunkley et al., 2004, 2006; Nikolovski et al., 2012), whilst the FFE approach identified proteins in fractions of purified Golgi, that were estimated to be enriched in medial Golgi cisternae (Parsons et al., 2012a). Immunoisolation of compartments has recently been used to great effect in separating components of the TGN, enabling comparative proteomics at the sub-Golgi level (Drakakaki et al., 2012). Characterization of Golgi-enriched fractions has been attempted in various plant systems (Tanaka et al., 2004; Asakura et al., 2006; Mast et al., 2010), major, large-scale proteomic characterizations have exclusively occurred in the model plant *Arabidopsis thaliana*.

## AN OVERVIEW OF THE *Arabidopsis* GOLGI–TGN PROTEOMES

**FIGURE 1:**
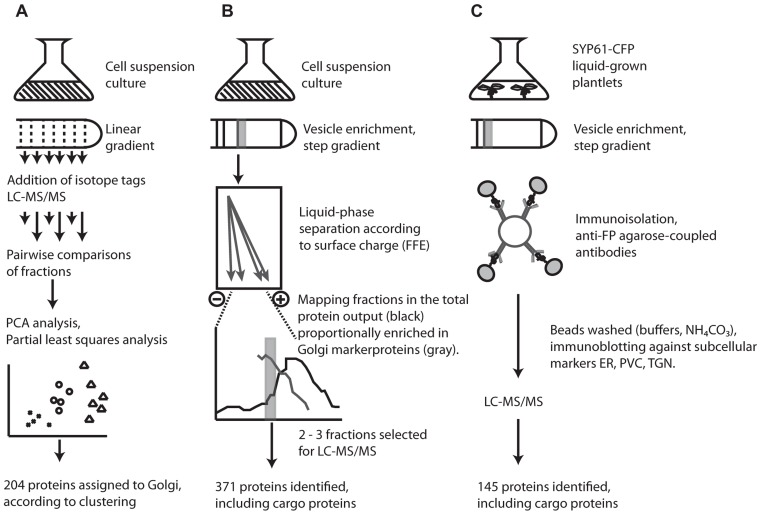
**Overview of the three different techniques employed in proteomic characterization of the *Arabidopsis* Golgi and TGN**. **(A)** Clustered proteins in LOPIT studies were assigned to the Golgi according to co-clustering with known and predicted Golgi marker proteins (for details, see Dunkley et al., 2004, 2006; Nikolovski et al., 2012). **(B)** FFE purified fractions were estimated at ca. 80% purity according to the proportion of previously localized Golgi proteins and contaminants present in each fraction; based on experimental data in SUBA (Heazlewood et al., 2007; for details, see Parsons et al., 2012a,c). **(C)** Isolation of SYP61 vesicles by affinity purification. Successful removal of contaminants during immunoisolation was assayed by the presence of the ER/*cis*-Golgi marker, BiP, and the prevacuolar compartment marker SYP21 (for details, see Drakakaki et al., 2012; Parsons et al., 2012c).

Initial attempts to characterize the *Arabidopsis* Golgi by mass spectrometry were undertaken nearly a decade ago with the aim of distinguishing between ER- and Golgi-resident proteins (Dunkley et al., 2004). The LOPIT approach involves quantitative mass spectrometry of proteins labeled with isotope tags. A cell homogenate separated along a linear gradient is fractionated and pairwise comparisons of fractions allow abundance ratios of isotope masses to be calculated for each protein. Proteins physically located in the same compartment will have similar ratios and so cluster together during partial least squares discriminant analysis (**Figure 1**). Using LOPIT, 89 proteins were initially localized to the Golgi (Dunkley et al., 2006) but the requirement that proteins carry all four tags limited the number of proteins for which a statistically credible localization could be assigned. Recent reanalysis and analysis of existing and new datasets, incorporating values for “missing” tags assigned using partial least squares regression models and training sets based on fully tagged proteins, enabled the collective localization of 204 proteins to the Golgi/TGN (Dunkley et al., 2006; Nikolovski et al., 2012).

Although a major motivation for the development of LOPIT was the difficulty in separating the Golgi, particularly from ER contaminants, a recent study has managed to isolate Golgi vesicles with an estimated 80% purity based on protein composition. This was achieved using a combination of sucrose density centrifugation and FFE (Parsons et al., 2012a). The power of FFE for organelle isolation was demonstrated in plants several years ago when applied to the separation of mitochondria and peroxisomes, two organelles which are typically hard to separate using density centrifugation alone (Eubel et al., 2008). As separation by FFE is dependent on surface charge, the Golgi, which carries a more negative surface charge than ER vesicles and most other contaminants, is amenable to separation using this technique, which resulted in 371 proteins being localized to the Golgi (**Figure 1**).

A dissection of the complexity of the Golgi proteome was recently attempted using immunoisolation of specific TGN trafficking populations. Affinity purified TGN compartments from plants expressing a syntaxin from plants (SYP61)-CFP construct were enriched for the TGN by sucrose density centrifugation then exposed to anti-FP antibodies coupled to agarose beads and analyzed by mass spectrometry (Drakakaki et al., 2012). Although widely used in mammalian systems, application of this approach in plants was precedential. The technique was able to identify 145 proteins from affinity purified samples of SYP61 vesicles, providing the foundation of a TGN proteome in plants.

## THE SIZE OF THE PLANT GOLGI PROTEOME

In total, 452 proteins have been characterized by mass spectrometry to the Golgi apparatus and 145 to the TGN from the model plant *Arabidopsis*. An ever-present question in subcellular proteomics concerns the total number of proteins present in an organelle. Given the residential/transitory definitions raised above, this is an especially difficult question to answer in the case of the Golgi and TGN, since proteins with ambiguous localization profile cannot be clearly assigned to a particular sub-compartment. Therefore dual-localized but Golgi-functional proteins or those at the *cis*-Golgi extremity will potentially be excluded from many analyses. Given the extensive subcellular localization data in the model plant *Arabidopsis* and the collection of subcellular prediction algorithms that are outlined in the SUBA database (Heazlewood et al., 2007), it is possible to make an estimation of the size of an organelle proteome based on an experimentally determined collection (Ito et al., 2011). Collectively, 491 proteins (excluding the defined cargo proteins) have been localized to the Golgi/TGN proteomes (Dunkley et al., 2006; Drakakaki et al., 2012; Nikolovski et al., 2012; Parsons et al., 2012a) and 145 proteins to the Golgi/TGN by fluorescent marker studies (Heazlewood et al., 2007). In total 575 unique proteins have been experimentally localized to the Golgi/TGN. Of the 22 subcellular prediction algorithms that have been applied to the entire *Arabidopsis* proteome, 14 provide a “Golgi” prediction output (**Table 1**).

**Table 1:** Estimated size of the *Arabidopsis* Golgi/TGN proteomes utilizing data in the SUBA database and the current integrated proteome (575) employing the abilities of subcellular prediction algorithms.

Predictor	Predicted Golgi *Arabidopsis*	Expt. any location (575)	Expt. in Golgi (575)	Expt. non-Golgi	FNR Golgi prediction	Est. correct predictions	FNR Golgi prediction	Predicted Golgi	Non-predictable expt. Golgi	Reference
AdaBoost	66	13	2	11	0.85	10	1.00	2919	2909	Niu et al. (2008)
BaCelLo	5178	1101	104	997	0.91	489	0.82	2704	2215	Pierleoni et al. (2006)
EpiLoc	290	121	30	91	0.75	72	0.95	1378	1306	Brady and Shatkay (2008)
iPSORT	5688	1439	177	1262	0.88	700	0.69	2273	1573	Bannai et al. (2002)
MultiLoc2	916	299	114	185	0.62	349	0.80	1762	1412	Blum et al. (2009)
Plant-mPLoc	1074	330	100	230	0.70	325	0.83	1871	1546	Chou and Shen (2010)
Predotar	5851	1438	186	1252	0.87	757	0.68	2340	1583	Small et al. (2004)
PredSL	8780	2082	259	1823	0.88	1092	0.55	2425	1333	Unpublished
PProwler	8885	2121	296	1825	0.86	1240	0.49	2409	1169	Boden and Hawkins (2005)
SLPFA	7738	1733	142	1591	0.92	634	0.75	2567	1933	Tamura and Akutsu (2007)
SLP-Local	7406	1599	167	1432	0.90	773	0.71	2663	1890	Matsuda et al. (2005)
TargetP	6492	1574	200	1374	0.87	825	0.65	2372	1547	Emanuelsson et al. (2000)
WoLF PSORT	112	45	11	34	0.76	27	0.98	1431	1404	Horton et al. (2007)
YLoc	1235	318	105	213	0.67	408	0.82	2233	1825	Briesemeister et al. (2010)

*Predicted Golgi *Arabidopsis*: All proteins predicted to be Golgi in *Arabidopsis* (TAIR10).*

*Expt. any location (575): Predicted Golgi and experimentally determined to be in any location by MS or FP (SUBA) or Golgi (575).*

*Expt. in Golgi (575): Predicted Golgi and experimentally determined to be in Golgi (575).*

*Expt. non-Golgi: Predicted Golgi but experimentally found to be non-Golgi [(Expt. any location) - (Expt. non-Golgi)].*

*FPR Golgi prediction: False positive rate for Golgi prediction. [(Expt. non-Golgi)/(Expt. in Golgi)].*

*Est. correct predictions: Estimation of correct predictions from total Golgi predictions in *Arabidopsis* [(Expt. any location) - (Expt. any location) × (FPR Golgi prediction)].*

*FNR Golgi prediction: False negative rate for Golgi prediction [1 - (Expt. in Golgi)/575].*

*Predicted Golgi: The predicted size of the proteome based on validated performance for each predictor program [(Expt. any location) × (1 - FPR)/(1-FNR)].*

*Non-predictable expt. Golgi: The size of the unpredictable Golgi proteome [(Predicted Golgi) - (Est. correct predictions)].*

Employing the relational capabilities of the SUBA database, it is possible to compute a size estimate of the Golgi/TGN proteome based on each algorithms performance. The overall performance of each prediction program can vary considerably with regard to the total predicted “Golgi” proteins in *Arabidopsis* (contrast AdaBoost, 66 Golgi and PProwler, 8885 Golgi) and positive prediction rate of the experimental proteome (contrast AdaBoost <1% and PProwler >50%). However, after calculating false positive and false negative rates for each program, the final predicted Golgi proteomes are remarkably similar. Based on this analysis, the *Arabidopsis* Golgi/TGN proteome is estimated to be 2239 ± 465, employing the average of the predicted proteomes of these 14 subcellular prediction programs.

## USING THE PROTEOME: WHAT ARE THE ROLES OF UNCHARACTERIZED PROTEIN FAMILIES?

A number of large gene families have been identified by both the FFE and LOPIT studies (Nikolovski et al., 2012; Parsons et al., 2012a). The quantitative mass spectrometry performed when applying LOPIT (Nikolovski et al., 2012) and spectral counts from FFE isolates (Parsons et al., 2012a), combined with localization data (Heazlewood et al., 2007), provide an important starting guide as to which members of these large families are major components and should be initially investigated in future studies.

The cyclophilin-like peptidyl-prolyl *cis*–*trans* isomerase family is consistently represented in the Golgi proteomes. These are known to catalyze conversion of *cis* to *trans *conformation of peptide bonds preceding prolyl residues in newly synthesized peptides (Chou and Gasser, 1997). In plants, they are classically associated with the thylakoid lumen where they are thought to help protein folding and assembly of photosystem complexes although their exact role is not clear (Ingelsson et al., 2009). The cyclophilins found by both FFE and LOPIT approaches (Nikolovski et al., 2012; Parsons et al., 2012a) localize either exclusively to the Golgi or are dually localized to the Golgi and plasma membrane (Dunkley et al., 2006; Benschop et al., 2007; Marmagne et al., 2007; Parsons et al., 2012a), implying a secretory-specific function, although no cyclophilins were found during immunoisolation of the TGN (Drakakaki et al., 2012).

The prenylated RAB acceptor B2 (PRA1.B2, AT2G40380) is found in both Golgi proteomes (FFE and LOPIT) but not the TGN, implying involvement with cisternal-specific interactions and vesicle docking. Examining proteins present uniquely in the TGN, besides those involved in trafficking such as the RAB GTPases, soluble *N*-ethylmaleimide-sensitive factor attachment protein receptors (SNARE; Blatt et al., 1999; Surpin and Raikhel, 2004), transport protein particle (TRAPP) components (Barrowman et al., 2010) or present as cargo, e.g., specific cellulose synthase A (CESA) subunits (Paredez et al., 2006), one endomembrane protein/transmembrane 9 protein (EMP/TMN9) and two *S*-adenosyl-L-methionine-dependent methyltransferases appear to stand out. Most EMP/TMN9 proteins are found in the Golgi cisternae: 11 members from a total of 12 were identified in FFE-purified samples (Parsons et al., 2012a) and 10 during LOPIT studies. EMP/TMN9 proteins interact with COPI and COPII proteins and membrane proteins destined for post-Golgi locations but are only recently studied in plants (Gao et al., 2012). The presence of two EMP/TMN9 proteins in both the Golgi and TGN implies *trans*-Golgi localization. With only one EMP/TMN9 identified uniquely in the TGN, members of the family may fulfill niche roles in trafficking depending on their location along the Golgi stack and are likely interesting subjects for future study. Apart from QUA2 (Mouille et al., 2007), a pectin methyltransferase in the *S*-adenosyl-L-methionine-dependent methyltransferases superfamily, no clear function has been assigned to any other members of this family of proteins in plants. The *S*-adenosyl-L-methionine-dependent methyltransferases which include QUA2 are prevalent in the Golgi and Golgi/TGN proteomes. A total of 20 were identified by LOPIT, 15 by FFE, and 3 in SYP61, resulting in 22 distinct proteins from this family (Drakakaki et al., 2012; Nikolovski et al., 2012; Parsons et al., 2012a). One member, AT5G64030, has been found in the plasma membrane proteome (Mitra et al., 2009; Zhang and Peck, 2011), so could conceivably function there. Assuming that all family members perform some kind of polysaccharide methylation, proteomic comparisons could be used to reveal late-acting enzymes in cell wall biosynthesis such as these examples.

Many functionally important Golgi proteins may actually be the sole members of their protein family. Of the 111 proteins not assigned to a functional protein category in the FFE proteome, 30 were also identified by LOPIT studies and many different protein families were represented. Amongst datasets such as these, dataset overlaps can provide a means to shortlist potentially important proteins about which little information is available.

Interestingly, although the proteomes comprised by the LOPIT studies and Parsons et al. (2012a) were both derived from similar starting tissues, a number of proteins are found in Parsons et al. (2012a) but not LOPIT studies and vice versa. Parsons et al. (2012a) identified more proteins overall and results included cargo proteins, unlike in LOPIT studies. Nevertheless after eliminating those annotated by Parsons et al. (2012a) as either transient or involved in protein synthesis, 81 proteins identified by LOPIT are not found in Parsons et al. (2012a) and 205 are in Parsons et al. (2012a) but not LOPIT. No clear pattern, e.g., protein abundance, exists between the proteins observed in either study; most probably differences arise from variations in methodologies, highlighting the value of multi-facetted approaches to proteomic characterization of the Golgi.

## WHAT IS MISSING FROM THE EXPERIMENTAL GOLGI PROTEOME?

Specific questions concerning what has not been identified so far are obviously difficult to answer but they can be addressed in part by examining what sorts of protein have been localized by fluorescent tagging but not identified by subcellular proteomic techniques. Fluorescent localization of proteins is generally motivated by interest in a specific protein and so is more likely to represent low-abundant polypeptides. It therefore provides an initial guide to the completeness of subcellular proteomic approaches.

Notably absent from proteomic surveys, but localized to the Golgi stack by fluorescent tagging are the Golgins and GRIP domain proteins (Latijnhouwers et al., 2007). Several glycosyltransferases such as cellulose synthase-like D5 (CSLD5; Bernal et al., 2007), rhamnogalacturonan II xylosyltransferase (RGXT) 1 and 2 (Egelund et al., 2006), irregular xylem 9 (IRX9; Pena et al., 2007), reversibly glycosylated polypeptide (RGP)1–4 (Drakakaki et al., 2006; Rautengarten et al., 2011), galacturonic acid transferase like (GATL) members from the GT8 family and a number of small GTPases are also either absent or poorly represented. Common methodological steps between these technically very different proteomes may in part explain these absences. Both the FFE and LOPIT approaches (Nikolovski et al., 2012; Parsons et al., 2012a) used cell suspension cultures whilst the immunoisolation approach (Drakakaki et al., 2012) used 14-day-old liquid grown plantlets as the starting tissue, meaning that all proteomes were based on primary cell wall-rich tissue. This may explain the absence of CSLD5 and IRX9, which are both implicated in secondary cell wall biosynthesis and localized to the Golgi stack (Bernal et al., 2007; Lee et al., 2007). RGXT1 and 2 may have been also have been missed because of tissue-specific or low expression (Egelund et al., 2006). Members of the GATL clade, although localized to the Golgi stack (Kong et al., 2011), are absence from all Golgi proteomes, which could point toward some specific spatial or temporal function of these glycosyltransferases. Golgins are Golgi matrix proteins with coiled coil domains that typically locate to the *cis*- and *trans*-extremities of the Golgi stack and cisternal peripheries. They are involved in regulation of stack architecture and tethering events during trafficking (Osterrieder, 2012). Their location to *cis*- or *trans*-extremities of the Golgi stack may have precluded detection (Nikolovski et al., 2012; Parsons et al., 2012a). Peripheral golgins and those with GRIP domains which localize to the TGN, have no predicted transmembrane domain and appear to be recruited from the cytosol by interactions with small GTPases. Their absence from either the Golgi or the SYP61 proteome (Drakakaki et al., 2012) may be due to carbonate washes used to remove cytosolic contaminants and/or centrifugation steps. Electron micrographs taken during FFE isolation procedure (Parsons et al., 2012a) show loss of vesicles from cisternal edges in with progressive centrifugation steps. Two of four data sets used in the LOPIT approach (Nikolovski et al., 2012) had been subjected to carbonate washes resulting in reduced peripheral proteins. This may explain why no RGPs have been detected, as these are peripheral membrane associated proteins (Delgado et al., 1998).

Several RAB GTPases have been localized by fluorescent protein assay to the Golgi stack (Batoko et al., 2000; Feraru et al., 2012). LOPIT approaches have identified two RAB GTPases localized to the Golgi, five were found by FFE purification (Parsons et al., 2012a) and 19 by immunoisolation (Drakakaki et al., 2012). RAB GTPases are involved in cargo-vesicle docking (Woollard and Moore, 2008) and are not Golgi-residents. This likely explains why fewer were present in the LOPIT Golgi proteome (Nikolovski et al., 2012). Step gradients employed prior to FFE purifications (Parsons et al., 2012a) were designed for maximal cisternal enrichment at the cost of small vesicles, so as to minimize ER contamination prior to FFE. This exemplifies the role of methodology in these technically diverse proteomes and shows how removal of contaminants may risk removal of Golgi-associated proteins.

Judging from these inconsistencies between the subcellular proteomics data and fluorescent protein localizations, it is clear that Golgi proteomics must be applied to other tissue types if the proteome is to be “completed.” This presents an even greater technical challenge as young, softer tissues are more easily homogenized to maintain Golgi stack integrity (Morre and Mollenhauer, 2009). However, useful information may be gleaned from less pure preparations using tougher, challenging tissue types, or preparations which are less pure but contains Golgi-associated and Golgi matrix proteins, as there is now a sufficiently broad base of proteins from which to compile ever more extensive markers and training sets.

## SUB-GOLGI PROTEOMICS AND THE GOLGI IN AN ENDOMEMBRANE CONTEXT

Comparative analyses such as those discussed above can now be formulated since a post-Golgi compartment has been characterized. The potential for distinguishing resident and cargo Golgi components can also be applied. Almost 30% of proteins identified in the TGN proteome comprise non-Golgi proteins as determined by the LOPIT approach (Drakakaki et al., 2012; Nikolovski et al., 2012). It is conceivable that with a few more post-Golgi compartments characterized, many of the endomembrane proteins currently assigned to multiple locations (Heazlewood et al., 2007) could be reassigned and more light shed on the various protein cycling routes through the secretory pathway. This could be reasonably achieved in a number of ways. For the smaller compartments such as endosomal compartments, the immunoisolation approach (Drakakaki et al., 2012) would hold the most promise as a number of syntaxin proteins known to associate with this compartment have been identified (Sanderfoot and Raikhel, 1999). Such an approach may not be appropriate for isolating individual cisternae from the main stack as trafficked proteins destined for later cisternae and TGN may also be detected by antibodies, whilst stack architecture could prove too complex for such an approach. Several fractions containing a high proportion of known Golgi proteins were not included in the FFE proteome owing to slightly higher level of contaminants. The number of fractions in which over 25% of proteins had been localized to the Golgi by LOPIT studies suggest partial electrophoretic separation of cisternae may have been occurring during the isolation process (Parsons et al., 2012a,b). A collection of sub-Golgi markers have been characterized (Saint-Jore-Dupas et al., 2006), so if proteins from FFE fractions could be accurately quantified profiles of co-migrating proteins could be created to enable sub-Golgi differentiation.

## CONCLUDING REMARKS

Although one of the most technically challenging organelles to isolate, a diversity of technologies have led to two Golgi proteomes and one proteome of TGN vesicles, resulting in nearly 500 proteins now localized to the Golgi and/or TGN by mass spectrometry. As the hub of protein trafficking, its proteome is best understood within the context of other proteomes; comparisons between these compartments bring a new level of understanding to protein distribution through the endomembrane system and show the potential for expansion through proteomic analysis of other post-Golgi compartments. It is estimated here that only about 20% of Golgi proteins have been identified thus far by mass spectrometry. So far all studies have been carried out in rapidly dividing, developing tissue (either cell suspension culture or liquid-grown plantlets). Exploration of other tissue types is needed to increase the coverage of the Golgi proteome. Efforts must also be concentrated in getting the proteomes of *cis*-, *medial*-, and *trans*-Golgi sub-compartments and specific vesicle populations. This will incur further technical challenges but will help identify more lowly expressed proteins and provide invaluable insight into plant Golgi functions.

## Conflict of Interest Statement

The authors declare that the research was conducted in the absence of any commercial or financial relationships that could be construed as a potential conflict of interest.
